# Health Agency and Perfectionism: The Case of Perinatal Health Inequalities

**DOI:** 10.1093/phe/phab009

**Published:** 2021-05-24

**Authors:** Hafez Ismaili M’hamdi, Inez de Beaufort

**Affiliations:** Erasmus University Medical Center Rotterdam

## Abstract

Poor pregnancy outcomes and inequalities in these outcomes remain a major challenge, even in prosperous societies that have high-quality health care and public health policy in place. In this article, we propose that justice demands the improvement of what we call the ‘health agency’ of parents-to-be as part of a response to these poor outcomes. We take health agency to have three aspects: (i) the capacity to form health-goals one has reason to value, (ii) the control one perceives to have over achieving those health-goals and (iii) the freedom(s) one has to achieve those health-goals. We will moreover argue that this demand of justice can be best based on a perfectionist rather than neutralist method of justification. Subsequently, we will argue that perfectionist policy may be paternalistic but not wrongfully paternalistic. This leads us to conclude that perfectionism should be adopted to inform and justify public health policy that is aimed at improving health agency in general and counteracting poor pregnancy outcomes and inequalities in perinatal health outcomes in particular.

## Introduction

There is a distinction between injustice and the perception of injustice, between how well life goes and how well one perceives life to go. The former expresses a state of affairs. In cases of injustice, this state of affairs is adverse. Inequalities in health, quality of life and life expectancy for instance, which are the result of an unfair distribution of societal opportunities, benefits and resources, are indicators of such injustice. The latter, however, expresses the ways in which life lived in unjust circumstances is perceived, if perceived at all, as an injustice. Infants that are born in unjust destitution for example, still lack the ‘means of perception’ to register their malnutrition, poverty and curtailed opportunities to live life well as matters of injustice. But even for adults who face unjust circumstances, it is not a given that they will perceive these circumstances as matters of injustice.

Take the case of perinatal health outcomes in a prosperous country with universal and subsidized high-quality health care such as the Netherlands as an example. On the one hand, the perinatal mortality and morbidity rates in the Netherlands are high compared to other European countries, especially those countries that enjoy similar prosperity (De Jonge *et al.*, 2013; Mohangoo *et al.*, 2014). Moreover, significant inequalities in perinatal health outcomes (IPH) exist. Research on the differences in perinatal health outcomes in the Netherlands has shown a staggering disparity in perinatal morbidity ranging from 5.2 per 1000 births in favorable geographic areas to 10.2 per 1000 births in unfavorable geographic areas (Waelput *et al.*, 2017). These poor pregnancy outcomes and the inequality in these outcomes have been labeled, repeatedly, as a matters of inequity and injustice as they are linked to undeserved medical and non-medical causes that are typically associated with life lived in underprivileged circumstances (Mackenbach, 2012; M’hamdi *et al.*, 2018a; Lagendijk *et al.*, 2019b;  Vos *et al*., 2014).

On the other hand, research has shown that women who have an increased risk to have poor pregnancy outcomes because of the unjust and underprivileged circumstances they live in, tend to not seek pregnancy-related care (M’hamdi *et al.*, 2017; Poels *et al.*, 2016; Goossens *et al.*, 2018). This tendency has been associated with: lacking information on pregnancy-related health and care, having a lower health literacy as well as the *perception* that they do not face increased risks for poor pregnancy outcomes (M’hamdi *et al.*, 2018b). In other words, life in underprivileged circumstances is not only associated with a higher risk to have poor pregnancy outcomes (Vos *et al.*, 2014) but also with poorer ‘preceptory skills’ to experience these higher risks as such (Poels *et al.*, 2016; M’hamdi *et al.*, 2018b). Poverty-related pregnancy risks do not advertise themselves, and may remain hidden to the women most prone to them.

The problem of poor pregnancy outcomes and IPH warrants at least two responses. Firstly, although pregnancy-related care is available in the Netherlands more needs to be done to improve the availability and accessibility of this care, especially for vulnerable women living in underprivileged areas. In this essay, we will argue that improving the availability and accessibility alone is necessary but insufficient to meet the demands of justice. Therefore, secondly, we will argue that to meet the demands of justice, the health agency of vulnerable women who face high risks to have poor pregnancy outcomes should be improved.

To make this argument, this essay will proceed as follows. First, we present the case of poor pregnancy outcomes and IPH in prosperous societies. We take the pregnancy outcomes of the Netherlands as the paradigm example. We will focus on the city of Rotterdam as the largest IPH have been recorded there. We will use the insights from epigenetics and the Developmental Origins of Health and Disease paradigm (DOHaD) to describe the ways in which social misfortunes become biologically impinged and consequently lead to avoidable poor pregnancy outcomes and IPH.

Subsequently, we will argue that poor pregnancy outcomes should be addressed by promoting and securing sufficient ‘health agency’ of parents. In fact, the demands of health justice should be concerned with the promotion and securing of health agency of all who fall within its purview. Two steps are required to make this point. First, the concept of health agency needs to be developed. This will be done by fleshing out its three aspects: (i) the capacity to form health-goals one has reason to value, (ii) the control one perceives to have over achieving those health-goals and (iii) the freedom(s) one has to achieve those health-goals.

Second, it has to be demonstrated why the demands of health justice apply to health agency. That is, we will explain why health agency is one of the appropriate focal metrics of health justice. To do so, we will couch the concept of health agency in the capability approach which has a formidable track record of demonstrating why agency—what people can do and be—is an appropriate focal metric of justice.

We will however, depart from the capability ‘canon’ in one important way. In general, to meet the demands of justice, the state has to promote and secure certain goods. In the capability approach these goods are the relevant capabilities. Given the wide array of capabilities, from trivial to those essential to human flourishing, decisions have to be made about which capabilities should be promoted as a matter of justice. Most capabilities scholars have argued that the state should promote and secure only those capabilities that promote lives worth valuing in a non-controversial way. That is, in choosing the relevant capabilities, the state should remain neutral on what ‘a life worth valuing’ entails. Consequently, the state should only promote and secure those capabilities that pertain to aspects of life that all reasonable persons to whom these capabilities apply, would value. This is known as the doctrine of neutrality. Most eminent capabilities scholars such as Sen and Nussbaum are staunch neutralists (Nussbaum, 2011). The improvement of health agency (but also a host of important capabilities) as we propose however, is controversial and would require non-neutral policy. By controversial we mean that reasonable well-willing individuals may disagree about whether promoting and securing sufficient health agency is a necessary precondition for living a life one has reason to value and thus whether it is a demand of justice.

Neutrality as a basis for health justice is attractive as it takes the right people have to live their lives according to their own lights seriously. It presumably strikes an appropriate balance between societal and personal responsibility for health. Despite the attractiveness of the neutrality doctrine however, issues of health justice can, in our view, hardly proceed along sensible lines if controversial judgments about health and wellbeing are eschewed. In other words, neutrality remains too quiet on disquieting issues such as poor pregnancy outcomes and IPH. This we find unsatisfactory. As a response, we will present our account of ‘simple perfectionism’. According to this view, there is an array of goods that make human lives go better. These goods are objectively determinable that is, the value of these goods is independent of the value that particular persons place on them. Given that the state has the duty to aid citizens to lead good lives, it therefore has the duty to promote and secure these objective goods. For health justice, one of these goods is, as we will argue, health agency. The state that espouses simple perfectionism should thus seek to advance the health agency of its citizens and of vulnerable mothers-to-be in particular.

Perfectionism as a political concept however, is suspect. The most important alleged moral defect of perfectionism is that it is wrongfully paternalistic. The perfectionist is indeed motivated by the concern for the wellbeing of persons and acknowledges that circumstances can arise in which persons are less likely to choose and act in accordance with their own good. In the last section, we will explain why although simple perfectionism is paternalistic it is not wrongfully paternalistic. The conclusion we reach is that simple perfectionism should be adopted to inform and justify health policy that is aimed at improving health agency and counteracting poor pregnancy outcomes and IPH in particular.

## Poor Pregnancy Outcomes and the Underpinning Mechanisms

It is unfair that by dint of the circumstances in which they enter the world children run the risk to be deprived of good health and the fruits of good health. This unfairness is arguably more disquieting when it occurs in societies in which these circumstances are not shaped by unfortunate chance such as the destitute conditions in poor countries, but rather by amendable choice. The Netherlands for example, a prosperous country in which free and high-quality health care is readily available, has relatively high and persistent poor pregnancy outcome numbers compared to other European countries (Poeran *et al.*, 2011). In addition, inequalities in pregnancy outcomes between neighborhoods—especially in the city of Rotterdam—are alarmingly high. Research done by Poeran *et al.* (2011) shows that: ‘[In Rotterdam] [t]he neighborhood-specific perinatal mortality rates varied from 2 to 34 per 1000 births, for congenital abnormalities from 10 to 91 per 1000 births, for IUGR [measure for poor fetal growth] from 38 to153 per 1000 births, for preterm birth from 34 to 157 per 1000 births and for low Apgar [measure for physical condition of a newborn immediately after birth] score from 4 to 37 per 1000 births. The highest mortality rates were observed in deprived neighborhoods’ (Poeran *et al.*, 2011; Vos *et al.*, 2014; Waelput *et al.*, 2017). These numbers demonstrate the pernicious impact of neighborhood inequalities on the lifelong health of newborns.

Much needs to be done to ameliorate the conditions of parents living in underprivileged neighborhoods. Research has identified a number of ‘barriers’ to prepare for pregnancy as a source of poor pregnancy outcomes. Some of these barriers pertain to the corrosive conditions in which underprivileged parents live their lives. These range from low-income levels, poor housing, air and noise pollution to maternal stress and domestic violence (Genereux *et al.*, 2008; Hobel *et al.*, 2008; Shah and Shah, 2010). To improve pregnancy outcomes in underprivileged neighborhoods, policy that addresses these corrosive conditions is of paramount importance.

Initiatives aimed at increasing the awareness of pregnancy preparation and the availability of pregnancy-related care have been launched (van der Zee *et al.*, 2011; Lagendijk *et al.*, 2019a; Sijpkens *et al.*, 2019). A striking example is the so-called ‘Healthy Pregnancy 4 All-2’ program which aims to identify vulnerable mothers living in underprivileged areas and young children at risk of adverse health outcomes to offer them pregnancy-related care (Lagendijk *et al.*, 2019a). This program has been initiated to address the challenge of delivering care to vulnerable women who are otherwise very hard to reach. Given the importance of a healthy pregnancy for the lifelong health of children, it is of great importance to address this challenge.

In fact, the period surrounding pregnancy is taking center stage in the endeavor to unveil the ‘origins of health and disease’ (Barker, 2004). The findings of David Barker in particular propelled research that focuses on the ways in which the impaired development of the fetus is linked to chronic diseases later in life (Barker, 1995; Barker *et al.*, 2002). This focus on the ‘Developmental Origins of Health and Diseases’ (DOHaD), ushered in a paradigm shift in which the importance of a healthy pregnancy for the lifelong health of newborns is demonstrated and espoused. The burdens of stunted fetal development are not only carried by newborns who become more prone to be born with congenital anomalies. A stunted fetal development entails a lifelong increased vulnerability to develop chronic diseases later in life such as cardiovascular diseases, certain types of cancer and type 2 diabetes. In other words, an impaired development in utero hits twice (Hanson *et al.*, 2011).

Research has shown that so-called ‘epigenetic mechanisms’ (partly) underpin the development of the fetus. Epigenetics is described as the mechanism that regulates the gene expression and thus to some extent health outcomes, without changing the DNA sequence (Holliday, 2006). An increasing number of clinical and epidemiological studies describe how preconceptional, prenatal and early life conditions of the fetus and newborn, which are related to the life conditions of the parents during this period, affect the epigenomic regulation of the gene expression and thus consequently the life-long health outcomes (Steegers-Theunissen *et al.*, 2009; Liu *et al.*, 2014). What is of particular interest is that the study of developmental processes and epigenetic mechanisms are increasingly elucidating the pathways through which social disadvantages become biologically impinged. Poor living environments, starting from the environment in utero, can turn into poor health. Although pathways such as aging, stochastic events and genotype are beyond human control, environmental and behavioral factors are to a large extent controllable. These factors include exposure to pathogens and pollutants, housing, work, nutrition and lifestyle (Ober and Vercelli, 2011). These factors are typically associated with parental socioeconomic status. The detrimental effects of having a lower socioeconomic status on the lifelong health of offspring are strongest during the period surrounding pregnancy (Messer *et al.*, 2015). In sum, substantial gains in lifelong health and wellbeing are achievable by adequate pregnancy preparation and the delivery of pregnancy-related care (M’hamdi *et al.*, 2018a). The lacking information, health literacy and perception of the urgency to adequately prepare for pregnancy are barriers to adequate pregnancy preparation. To address these barriers, we will argue that the promotion of so-called parental ‘health agency’ is necessary. To make this point, we will first develop the concept of health agency in the next section.

## Health Agency

We have stated that in underprivileged neighborhoods within prosperous societies, lacking information, health literacy and perception of the urgency to adequately prepare for pregnancy can, in tandem, impair the health agency of parents. But what exactly is being impaired when we claim that the health agency is impaired? To answer this question, we need to unpack the concept of health agency. So far, we have presented health agency as (i) the capacity to form health-goals one has reason to value, (ii) the perceived control over achieving those health-goals and (iii) the freedom(s) to achieve those health-goals. First, we discuss the capacity aspect.

## Capacity

A broad but uncontroversial definition of agency is: ‘the *capacity* to act intentionally’ (Schlosser, 2015). An agent is anyone who has this capacity. Actions are intentional when they are performed because of underlying reasons. Agency is thus the capacity to act in accordance with some underlying reason. This depiction of agency is strictly descriptive. What matters from a normative viewpoint is that the reasons one has to act, that is, the reasons one has to establish that a goal is worthy of pursuit through action, have to meet some standard of authenticity. These reasons have to belong in a meaningful way to the agent. Agency thus refers to the capacity to act on reasons (descriptive) aimed at achieving a goal one has reason to value (normative). For example, consider a diabetic person who fails to take her daily insulin shots. Imagine that she struggles all her life with basic organization. There is a reason for her failing to take her daily shots; she is unorganized. Let us assume however, that she does not have a reason to value being unorganized. That is, she has no reasons to value the reason for (in)action. Therefore, in terms of agency, the capacity aspect of her agency vis-à-vis taking daily insulin shots, is impaired.

This description of agency is sometimes equated with autonomy. Autonomy is a controversial and heavily debated concept. Explaining the capacity aspect of agency only in terms of autonomy therefore runs the risk of replacing one concept that requires clarification by another concept that requires clarification. Hence, we will further develop the concept of health agency without referring to autonomy even though we recognize the kinship of both concepts.

Coming back to the capacity aspect of agency, we propose one further qualification. We are interested in *health* agency. Consequently, we only take the health-goals of agents into consideration. This of course is not an innocent demarcation. One of the most important insights of epigenetics, DOHaD and the study of social determinants of health is that the circumstances in which people live their life, the overall goals they aspire to and the level of overall wellbeing they achieve are all factors that influence health. This entails that the sphere of non-health goals will always bleed into the sphere of health goals. The goal to become a lawyer for instance, is to some extent also health-related as the social stratum in which lawyers typically live their lives is typically associated with good health. At the same time, it would be inappropriate to understand one’s goal to become a lawyer—primarily—as a health goal. As a course-grained demarcation, we propose to use the label ‘health goals’ for those goals that people have to, above all, promote their health or the health of someone they are responsible for (such as their children).

In sum, the capacity aspect of health agency refers to the capacity persons have to form health-goals one has reason to value. The importance of this capacity condition for (health) agency is certainly not new and can be found in the capability approach-based literature (Ruger, 2007).

## Control

Control is typically considered to be an important condition for agency. When we act as self-governing agents who shape life according to our own will, we need to feel ‘in control’ of what we do and what happens to us. This experience of being in control, or the lack thereof, has been described in the psychological literature as the ‘locus of control’. Locus of control depends on the experience of individuals regarding the source of the attainment of a goal. The source of control can be internal or external (Lefcourt, 1991; Cobb-Clark *et al.*, 2014). This makes control an important aspect of (health) agency. An individual who attributes the success (such as having good health) in her life to the choices she made will gain confidence in her competence to successfully pursue health-goals worth valuing (such as bearing a healthy baby). In other words, she has an internal locus of control. There is evidence suggesting that women living in underprivileged neighborhoods experience limited internal control over their pregnancy and the health of their offspring (M’hamdi *et al.*, 2018b). Although this group of women is open to receiving help and care, they tend not to seek it because of their perceived limited control over their pregnancy and their pregnancy outcomes (Poels *et al.*, 2016). These insights serve as the basis for our second aspect of health agency: the perceived control over achieving health-related goals one has reason to value.

## Freedom

Capacity and control are aspects that apply to the ‘internal’ conditions of the agent. There are however, also ‘external’ conditions that are important to agency. These are the conditions that pertain to availability of the right set of liberties, opportunities and material goods which are required to realize a goal one has reason to value and one experiences sufficient internal control over. For example, a mother-to-be may formulate, as a result of her health agency capacity, the goal to adequately prepare for pregnancy. She therefore values taking folic acid. She might also experience the daily use of folic acid as a task within her control. Still, she might be limited in her liberty, opportunity or resources to buy folic acid. These external conditions hamper her ‘real freedom’ to achieve her health-goal.

In line with Sen, we argue that real freedom is an indispensable aspect of agency. Sen describes ‘agency freedom’ as ‘what the person is free to do and achieve in pursuit of whatever goals or values he or she regards as important’ (Sen, 1985). For Sen agency freedom consist of two aspects namely ‘control’ and ‘power’. The control aspect is already incorporated in our description of health agency. The power aspect of freedom is also indispensable for understanding health agency. Interestingly enough, power as an aspect of agency features frequently within the healthcare debate although in a slightly different guise. It typically appears as a claim about the importance of em*power*ment of individuals to improve their health (and the health of their offspring) (Wallerstein, 1992).

When it comes to health agency, we also endorse the idea of freedom as a power or as empowerment. We understand empowerment as the improvement of real freedom that pertains to what we have designated as the external conditions of agency. For example, to provide information and advice about the health benefits of pregnancy preparation and to make a healthy diet, supplementary vitamins and preconception care consultations readily available increases the real freedom women have with respect to their pregnancy. They become empowered.

The freedom condition of health agency thus refers to the real freedom to actually do what is necessary to achieve the health goals one has reason to value. Summing up, our last condition for health agency is 3. The freedom one has to achieve health-goals one has reason to value.

It is important to consider that although these three aspects (capacity, control and freedom) of health agency are conceptually distinct, they are interdependent. If one’s capacity to form health-goals one has reason to value is compromised this will likely adversely influence the perception of control and vice versa. As is often with conceptualization, conceptual demarcations are an important vehicle for the analysis of problems, yet they almost never do justice to the complexity of real life.

We have fleshed out the concept of health agency. The next step is to explain why health agency is the appropriate (but not sole) good that should be promoted and secured as a matter of health justice. More specifically, given that our initial aim was to counteract poor pregnancy outcomes and IPH, one may wonder what makes the focus on health agency as a demand of justice apt to achieve said aim.

To answer these questions, we will first give a short description of the capability approach and explain why our concept of health agency should qualify as one of the goods that should be promoted and secures by the demands of health justice. We are aware that the selection of the capability approach as the appropriate approach to cash out the demands of justice is not self-evident. Disputes about the appropriate approach or theory of justice are pervasive and intractable. Still, the capability approach is a serious contender in the debate about which theory of justice does justice to justice. Those who see serious flaws in the capability approach might also see them in our rendition applied to health justice. A discussion on which theory of justice is most worthy of allegiance is however, beyond the scope of this article. We will end the next section with an explanation of why the promotion of health agency is apt for counteracting poor pregnancy outcomes and IPH.

## The Capability Approach, Health Agency and Reasonable Disagreement

In short, the capability approach is a normative framework with which the demands of justice vis-à-vis social and political arrangements can be assessed. Whether a social or political arrangement is just depends on the extent to which individuals have substantial freedoms ‘to do and be what they have reason to value’ (Robeyns, 2011). These freedoms to achieve goals one has reason to value are called capabilities. Sen, argues that ‘one’s freedom to achieve those things that are constitutive of one’s own well-being’, is one’s set of capability (Sen, 1992). In other words, one’s real freedoms to promote one’s well-being can be understood as the sum of one’s capabilities. Crucial for our argument is the distinction that Sen makes between freedom that pertains to one’s own wellbeing and freedom that pertains to one’s agency. Sen argues that one can have reasons to value goals other than the sole promotion of one’s own wellbeing. Parents for example, typically pursue the promotion of the health and wellbeing of their children even if it comes at some cost to themselves. Sen calls the freedom to pursue goals that are not only restricted to the improvement of one’s own wellbeing *agency freedom.*

Based on this distinction, we label the good we seek to promote (the capacity, control and freedom triad) as health *agency* rather than, say, health capability. That is, given that we are dealing with poor pregnancy outcomes, the capacity, control and freedom that parents have to avoid preventable poor pregnancy outcomes, which will benefit their offspring, is better described as a matter of agency freedom rather than as a matter of capability.

Moreover, as we are discussing the capacity to improve the *health* of persons such as newborns *health* agency seems to be most apt description of the metric of justice we have in mind. Still, the normative logic of the capability approach applies to the promotion of health agency in the name of health justice. According to the capability approach, the real freedoms that people have to be or do those things they have reason to value is what makes life go better. Therefore, justice demands the promotion and securing of those real freedoms. Within the domain of health, the health agency of persons to make health-decisions they have reasons to value is what makes life go better. Therefore, health justice demands the promotion and securing of health agency. Given that we are dealing with poor pregnancy outcomes and IPH one may wonder whether the focus on health agency is apt for this specific case. We think it is for two reasons. First, some of the barriers to adequate pregnancy preparation are agency-related such as lacking information, low health-literacy and a lacking perception of the pregnancy-related risk vulnerable mothers-to-be face. These barriers curtail the capacity women have to make authentic self-governed decisions pertaining to their pregnancy and the health of their children-to-be. Thus, restoring the health agency should amount to better pregnancy outcomes. Second, although health agency includes the liberty to make one’s own autonomous health-related choices this liberty is not unbridled. Parents-to-be who have a desire to have children, typically have—and if not, they should have—the desire to have children who enjoy a reasonable quality of life. Most parents actually strive for more than reasonable quality and want the best for their children. That is, parents *qua parents*, want what is reasonably good or even what is best for their children. When parents make decisions (or fail to make decisions) that would very likely put their children at the risk of living a life below the threshold of a reasonably good quality of life, these decisions are ‘red flags’. They can, for one, be indicators of agency problems; problems with the capacity, the control or the freedom to make decisions that redound to the benefit of their children. Again, investing in the improvement the health agency of these parents should be at the very least part of the solution to this problem. If it is the case however, that parents willingly, that is—as an expression of their agency—make decisions that would very likely to put their children at the risk of living a life below the threshold of a reasonably good quality of life then the state has a good reason to override parental health agency. Paternalistic policy or ‘prevention of harm to others’ policy is apt in these situations. In many countries, these policies, such as law and policy against child abuse, exist. This demonstrates that even though health agency of parents is paramount it certainly is not the only consideration when it comes to the duties of the state to promote and safeguard the health and wellbeing of its citizens and its youngest citizens in particular.

The assertion that justice demands the promotion of health agency is of course not uncontroversial. Reasonable disagreement is possible about whether justice does in fact demand the improvement of health agency. If one is, for example, committed to a more libertarian view, the available opportunities to seek pregnancy-related care (such as those available in the Netherlands) are more likely to be sufficient to satisfy the demands of health justice. Making the improvement of parents’ health agency a matter of health justice would, in that view, be taking the societal responsibility for health too far and allow for too much state interference in the lives of people in the name of justice. The capabilities approach faces a similar challenge of reasonable disagreement. There are countless capabilities imaginable, from the capability to tie one’s shoe laces to the capability to express one’s political views. Given the wide array of capabilities, from trivial to essential, the question arises which capabilities should be promoted as a matter of justice. Similar to the health agency challenge, the selection of capabilities that should be promoted as a matter of justice can also give rise to reasonable disagreement.

In cases in which reasonable disagreement with respect to the demands of (health) justice arise, two responses are typically given, at least in the field of political philosophy (Ackerman *et al.*, 2003). First is to argue that, whatever the demands of justice are, the state and its institutions have a duty to remain neutral on issues about what a good life for citizens entails. Given that justice is concerned with the promotion and securing of lives worth valuing, it follows that the state and its institutions should limit themselves to formulating (health) policy that promotes and secures non-controversial that is, neutral goods. This is known as the neutrality doctrine (Gaus, 2003). Consider for example, that whatever theory of justice one adheres to, down the line all can reasonably agree on the assertion that the availability of basic health care makes life go better. Making basic health care available is therefore a neutral demand of justice. Capability scholars are typically neutralists.

The second response is to deny that the state has this stringent duty to remain neutral (Arneson, 2000; Ackerman *et al.*, 2003). This is the non-neutrality or perfectionist doctrine. We will argue in favor of perfectionism. More specifically, the view we have in mind is that: (a) there is an array of goods that make human lives go better which are objectively determinable and whose value is independent from the attitudes persons have toward them and (b) the state has a duty to aid citizens to lead good lives and therefor a duty to make those goods that make life go better available (and those things that make life go worse unavailable or harder to get). Let’s call the view that accepts (a) and (b), simple perfectionism.

The simple perfectionist rendition of health justice entails that when it comes to health, health agency is valuable. It is valuable to such an extent that the state and its institutions have the duty to help citizens promote and secure health agency. Moreover, the value of health agency is independent of individual valuations of health agency. That is, all things being equal, citizens qua citizens live objectively better lives when they have more rather than less health agency. This certainly does not entail that citizens do not or should not value health agency. It is only to say that sufficient health agency makes one’s life go better. This moral claim can give rise to reasonable disagreement. But unlike the neutralist, simple perfectionists, see reasonable disagreement as a reason to start rather than to close the discussion on what justice demands. To be sure, perfectionism and the advancement of agency are not necessarily antagonistic. There is a long tradition that is labeled ‘liberal perfectionism’ which goes back to J.S. Mill and which is espoused by contemporary scholars such as Joseph Raz, Steven Wall and Richard Arneson in which it has been argued that perfectionist policies are a necessary condition to put citizens on the path of leading an authentic self-governed life. Mandatory education for children and adolescents as well as having robust public health policies in place are examples of perfectionist policies that help citizens to live autonomous lives. The former is necessary to develop the capacity to make autonomous choices and the latter is necessary to prevent diseases which have debilitating effects on one’s capacity to pursue one’s self-determined aims in life. Being severely ill and bed-ridden because of COVID-19 for example, curtails one’s capacities and opportunities to realize one’s life plans. In other words, perfectionism is not the antagonist of but rather a prerequisite for (health) agency.

## Perfectionism and Neutrality

Perfectionism has been attacked quite fiercely by political philosophers who maintain that the state has a stringent duty to remain neutral regarding questions of the good (Quong, 2011). According to political neutralists such as John Rawls, Martha Nussbaum and Jonathan Quong, the exercise of state power, even to promote valuable goods, is only legitimate when it is justified by appeal to principles that all reasonable persons subject to this exercise of power can accept (Rawls, 2005; Nussbaum, 2011). Given that reasonable persons will differ, and differ widely, about which goods are valuable to them, the state should refrain from making these judgments. Consequently, the state should also refrain from formulating controversial policy. Given that the perfectionist rendition of justice allows for controversial judgments about the good, it fails to respect reasonable persons as free and equal individuals and therefore perfectionism is morally unacceptable, or so the argument goes.

According to the neutrality doctrine, the legitimacy of institutions that formulate health-related policy depends on whether the rationale that underpins this policy can be justified to all subject to it, each from their own evaluative perspective. This criterion that demands legitimacy on the part of the state and its institutions and reasonableness on the part of citizens has been the subject of much discussion. For one, this criterion is conceptually speaking too nebulous for a criterion that carries this much justificatory weight. It remains for instance, unclear on which grounds citizens may reasonably disagree with the principles underpinning some policy that is being put forward by the state. Nussbaum claims that only normative grounds count. Rawls and Larmore allow for some epistemic grounds (Larmore, 1987; Nussbaum, 2011). Still, what makes disagreement *reasonable* remains arcane. We will not further discuss this problem but for the sake of argument simply assume that it is clear what legitimacy and reasonableness demand. Nevertheless, the doctrine of neutrality, especially on issues of health justice, remains problematic.

When addressing problems such as avoidable poor pregnancy outcomes and the staggering inequalities in these outcomes, controversy about the course of action that ought to be taken is bound to arise. Consequently, the neutral state is barred from formulating health policy to counteract these poor pregnancy outcomes as the neutral state must remain neutral with respect to controversial issues. Take for example, the balance between the demands of health justice on the one hand and the parental responsibility for the health of their offspring on the other. There are many reasonable ways to strike the balance between these two considerations. Given the intractable disagreement on the balance between personal and societal responsibility within the justice discourse, waiting for consensus is tantamount to waiting for Godot. Because of the reasonable disagreement on this matter, the state that adheres to the neutrality doctrine is barred from formulating policy to counteract this problem. This bar on controversial policy making however, is unsatisfactory. If all controversial issues of justice are eschewed, the aim of justice—to promote and secure the goods and conditions for a good life—will be forfeited. Perhaps it is possible to satisfy the demands of neutrality and reach consensus on policy that safeguards some minimal opportunity to seek pregnancy-related care. This however, would remain an unsatisfactory outcome. Pregnancy outcomes such as those in Rotterdam show that (suboptimal) opportunities to seek care alone do not suffice for parents who live in underprivileged areas. Initiatives such as the Mothers of Rotterdam program go beyond offering opportunities and provide actively aid to vulnerable mothers, for example, by providing care at the home as well as accompanying them in their visits to see health care professionals (Van Der Hulst *et al.*, 2018). This is an apt example of perfectionist policy. To put our criticism of neutrality in general terms: the upshot of neutrality is that either controversial health issues are barred altogether or health-related policy is formulated and watered down to such an extent that it becomes uncontroversial but also unimpactful. Both outcomes are unsatisfactory.

We therefore propose to adopt an objective list approach to our account of simple perfectionism. In short, the objective list ‘theory’ is a theory of value which holds that there are basic goods, for example, health agency, security and the opportunity to receive medical assistance, which are intrinsically valuable to everyone (Parfit, 2012). The goods on this list should be promoted as a matter of justice. The goods on the list are pluralistic. They may include liberties, opportunities, real freedoms, virtues and material goods. The list is objective because the value of these goods does not depend on whether they are actually desired by persons that are entitled to receive them. One’s life goes better when one has more goods on the list. The identification of the basic goods on the list can proceed along the same lines as the identification of basic capabilities or the identification of opportunities worth equalizing in a fair manner. That is, the identification of the basic goods can be based on public deliberation that is guided by the method of reflective equilibrium. Or if you will, the items on the list are determined by careful deliberation on which things make life go better. The list is open ended and revisable.

This is but a sketch of the objective list approach. It should be noted that there are more theories of value, the most noteworthy are desire satisfaction and mental state theories. The benefit of using an objective list approach is that it enables objective interpersonal comparisons. All things equal, someone who has sufficient health agency is better off than someone who has insufficient health agency. Moreover, once a controversial item such as health agency appears on the list, justice demands its proper distribution. In the following section, we will further develop our account of simple objective list perfectionism by addressing its strongest objection; the alleged wrongful paternalism.

## Perfectionism and Its Discontents

Is perfectionism wrongfully paternalistic? Perfectionism does allow for controversial health policy. For the neutralist, however, allowing for controversial health policy amounts to the state failing to respect its citizens as free and equal. That is, citizens are not treated as free for their liberty and autonomy are restricted through policy, for their own good. And citizens are not treated as equals for health policy can be based on controversial ideas about what is of value thereby favoring the ideas of one group of citizens over another. Before addressing these concerns, it is important to take note of what paternalism in the context of justice entails. Consider our following proposition that is based on the work on paternalism done by Shiffrin (2000). The state and its institutions act paternalistic when they formulate liberty or autonomy-limiting policy based on the concern for the wellbeing of some group of individuals in the context of some decision, which lies within their legitimate control, coupled with a negative judgment of the ability of that group of individuals to make wise or prudent decisions in that context.

It is important to note that we limit our account of simple perfectionism to health justice. That is the context in which the state formulates health policy which is based on its judgment of the ability of persons to make wise or prudent decision. When determining the wrongfulness of paternalism, this context matters greatly. In the context of—say—political rights and freedoms and matters of belief, perfectionism is hard to defend. Judgments of the state and its institutions about the soundness of the ideas, values and convictions that people hold on these matters are inappropriate as they touch on the deepest and most comprehensive commitments that persons have. These commitments are tightly bound up to people’s sense of who they are. Therefore, the state’s judgments and consequent controversial policy are more likely to be wrongfully paternalistic in these contexts. The deeper policy touches citizens’ identities the stronger the case for neutrality.

But there is a difference between judgments about citizens’ ideas and values regarding political issues, friendship, love and God and judgments about citizens’ ideas and values regarding public transportation, television broadcasting and public health. That is, although all these matters may touch citizens’ identities, the extent to which they do varies and this ‘identity embeddedness’ can be placed on a continuum. The former matters touch citizens’ identities stronger than the latter. Therefore, controversial state policy is less appropriate in the former sphere than it is in the latter.

For instance, the fact that parents living in underprivileged conditions typically do not appraise themselves as having higher risks to have poor pregnancy outcomes is not likely to follow from a commitment that is tightly bound up to the sense of who they are. It is very unlikely to be constitutive of their identity. Therefore, the claim that health-related policy that aims at improving health agency so to improve pregnancy outcomes is disrespectful as it denies the freedom and equality that is owed to all citizens, seems exaggerated. In fact, the antithesis seems more plausible. Policy that encourages the promotion of parents’ health agency may help them to make health-related decisions that better reflect the goals and values they have reasons to hold, that is, that express rather than deny the sense of who they really are. In this way citizens are being respected as free and equal.

This requires health policy that takes seriously the fact that like all other people, parents can have deficiencies in their capacity to pursue their good especially when they live in underprivileged conditions. By the same token health policy should also take seriously the fact that parents can reflect about their health-related choices and revise them when they are presented with reasons to do so. All this involves not shying away from paternalistic policy. It involves formulating policy that, given the goals of parents qua parents, expresses the value judgment that for example, it is objectively better to stop smoking before and during pregnancy. We see no reason to remain neutral on that issue.

Of course, it is paramount that controversial health policy is based on sound empirical insights as for example found in SDH research and widely shared values. But, with the appropriate empirical and normative baggage on board, the state can respect parents qua parents as free and equals even when it seeks to revise their health-related preferences and goals and improve their health agency through perfectionist policy. In other words, simple perfectionism applied to health justice, is paternalistic but not wrongful.

We would like to end with a caveat. The fact that persons can have impaired health agency offers no license to the state to engage in formulating unbridled liberty and autonomy-limiting health policy. The adherence to values such as proportionality and subsidiarity should result in the requirement to use state power to intervene in the lives of people in a proportionate manner and the requirement for the state to always seek the least intrusive means to achieve health goals. As with all policy, a balance needs to be struck between the burdens that policy brings about and the good it seeks to promote. This is no easy task. Arriving at fair health policies is challenging. But eschewing controversial health policy amounts to giving up the pursuit for fair health policy altogether.

## Conclusions

We have argued that heath justice demands the improvement of health agency of persons and parents living in underprivileged conditions in particular. Simple objective list perfectionism provides the most promising normative approach to formulate health policy that satisfies these demands.
